# Association and Expression of Virulence from Plasmids of the Group B Strain in *Pseudomonas syringae* pv. *eriobotryae*

**DOI:** 10.3390/pathogens7020041

**Published:** 2018-04-14

**Authors:** Tran Dang Khanh, Tran Dang Xuan

**Affiliations:** 1 Agricultural Genetics Institute, Pham Van Dong Street, Hanoi 122300, Vietnam; khanhkonkuk@gmail.com; 2 Division of Development Technology, Graduate School for International Development and Cooperation (IDEC), Hiroshima University, Higashi Hiroshima 739-8529, Japan

**Keywords:** *Pseudomonas syringae* pv. *eriobotryae*, pigment, pathogenic, *psv*A, virulence gene

## Abstract

*Pseudomonas syringae* pv. *eriobotryae* causes serious stem canker in loquat (*Eriobotrya japonica*) trees. This study was conducted to determine whether plasmids are involved with its virulence. The strain NAE89, which belonged to the B group, harbored two plasmids at approximately 6.2 and 50 Mdal that caused stem canker and halo leaf spots on loquat plants. Following digestion with *Bam*HI and ligation into the *Bam*HI cloning site of the broad range host cosmid pLAFR3, four DNA fragments at 3.8, 6.6, 12.3, and 22.8 kb were generated. Although the plasmid-encoded virulence gene *psv*A was undigested with the *Bam*HI, the halo leaf spot gene may be adjacent to the *psv*A gene was digested. A pLAFR3 cosmid clone was introduced into the non-pathogenic PE0 and NAE89-1 strains by triparental matings and the pathogenicity was recovered. As a result, the pLAFR3 cosmid clone was introduced into the largest size DNA fragment of 22.8 kb and determined to be the causal agent of canker on the stem of the loquat. This study revealed that the *psv*A gene, previously found in the 50 Mdal plasmid, was also observed in the 22.8 kb DNA fragment.

## 1. Introduction

*Pseudomonas syringae* pv. *eriobotryae* is a pathogenic bacterium that causes stem canker in loquat trees. The pathogen attacks all plant parts, causing weak growth and reduction of commercial value of fruits [1,2]. Damage from this disease has been documented in Argentina, Australia, China, Japan, New Zealand, and the US [3,4,5,6,7,8,9]. The stem canker bacterium has been classified into three groups—A, B, and C—based on their pigment production and pathogenicity to loquat leaves [10]. The A and B strains do not produce pigment. Unlike group A strains, group B strains are pathogenic to mesophyll. The group C strains produce dark brown pigment, but are not pathogenic to mesophyll [11,12]. However, all of the three bacterium groups cause pathogenicity to the stems of loquat plants. In particular, the group B strains cause both stem canker and halo leaf spot. 

The strain NAE6 was found to belong to the A group of *Pseudomonas syringae* pv. *eriobotryae* (*Ps.* pv. *eriobotryae*). While it contained the 25, 52, and 60 Mdal plasmids, the 52 Mdal plasmid appeared to be required for virulence [13]. A pLAFR3 cosmid clone, which contained a 23 kb inserted DNA derived from the 52 Mdal plasmid, was designated the pVIR6 and reintroduced into a cured virulent PE0 strain with the helper plasmid pRK2014 [13]. The conjugates that received pVKR6 regained virulence. It was proposed that the 52 Mdal plasmid possessed the virulence gene(s) [13]. In the group A strains, a loss of the 85 Mdal plasmid was associated with the disappearance of virulence. Attempts to reintroduce the 85 Mdal plasmid into a cured strain have not been successful [13]. 

Several studies were conducted to determine the involvement and expression of plasmids with regards to virulence gene(s) such as the halo bright pathogen of bean, *Pseudomonas savastanoi* pv. *phaseolicola*, which consists of nine races, eight of which harbored large plasmids of about 150 kb. The pAV511 resulted in the loss of virulence in bean cultivars [14]. A similar native plasmid for curing of an approximately 82 kb native plasmid by heat shock (32 °C) from a strain of *Ps. syringae* pv. *eriobotryae*, the causal agent of stem canker in the loquat, led to a failure of pathogenicity in the host plant [13]. A single gene, *psv*A, identified by Hiehata et al. [15] and named *Pse-a*, was capable of restoring pathogenicity [13,15]. The gene, with a low % G + C and potential *hrp*L promoter sequence, showed no similarity to other genes in the databases. An exception was a small portion of the N-terminus of the putative protein products, which had a similarity to AvrA [16]. Many plant pathogens, particularly the soft-rot pathogen *Erwinia carotovora* and some pathovars of *Xanthomonas campestris*, secreted extracellular enzymes that were specific to chromosomal genes [17]. The others included the pathogen *Burkholderia cepacia* and some plant strains cause bulb-rot of the onion (*Allium cepa*) [17].

The production of an *Endopolygalacturonase* strain was found to be due to the gene, *peh*A, which was located on a 200 kb plasmid [18]. A potential *vir* gene, designated *virPphA*, restored virulence to bean when introduced into the cured race 7 strain RW60 [18]. However, it conferred a virulence to certain soybean (*Glycine max*) cultivars. Given the common regulatory features of *virPphA* and the *avr* genes in *Ps. syringae*, implied that they might be the virulence genes [18]. A gene showing dual *avr*/*vir* gene behavior was isolated from the pPATH plasmid of *Erwinia herbicola* pv. *gypsophilae* [19]. 

It was reported that the resistance to loquat canker (group A) controlled by a single dominant gene, designated as *Pse-a* [15]. The wild species bronze loquat (*Eriobotrya deflexa*) and was mapped [12] to reveal the presence of *Pse-a*, with the constructed linkage group spanned by 69.4 cM and had an average marker density of 2.6 cM [12]. Resistance to the group C bacteria was controlled by a recessive gene *Pse-c* [20], and mapped [2]. Hiehata et al. [21] screened 52 loquat cultivars for resistance to groups A, B, and C. There were 25 resistant cultivars, whereas 22 were susceptible to group A. Most of these observations exhibited similar responses to group B, thus it was suggested that the resistance of the gene *Pse-a* might be associated with groups A and B [21]. However, further pathogenic analyses on group B have not been carried out. 

Although much attention has been focused on the A and C strain groups of the *Ps.* pv. *eriobotryae* as mentioned above, little information was available on the B strain group, which uniquely caused both stem canker and halo leaf spot (Supplement 1). In addition, the involvement of the pathogenic gene(s) involved in the B strain groups has remained unknown. Hence, this study was conducted to investigate the pathogenic expression of the plasmids in the B strain group. 

## 2. Results

### 2.1. Cloning Fragments from Plasmid NAE89

**Figure 1:**
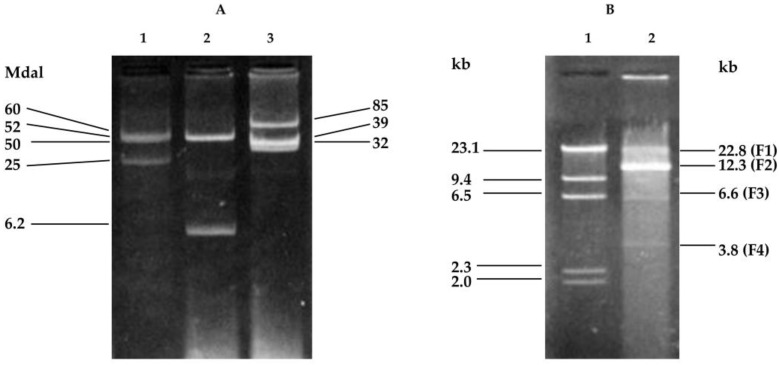
(**A**) Agarose gel electrophoresis of the plasmids from *Ps.* pv. *eriobotryae*; Lanes 1: 60, 52, and 25 Mdal plasmids of NAE6 (group A); Lanes: 50, 6.2 Mdal plasmids of NAE89 (group B); Lanes 3: 85, 39, 32 plasmids of NAE57 (group C); (**B**) Agarose gel electrophoresis of NAE89 plasmids digested with *Bam*HI. Lane 1: λ/*Hin*dIII, 23.1, 9.4, 6.5, 2.3, and 2.0 kb. Lane 2: NAE89/*Bam*HI with 22.8 (F1), 12.3 (F2), 6.6 (F3), and 3.8 kb (F4).

Plasmids of the NAE89 strain were extracted following a method described in Kado and Liu [22], and used for agarose gel electrophoresis. Similar plasmid groups were extracted, including the NAE6 bacteria (A group) and NAE57 bacteria (C group). The NAE89 strains harbored two kinds of plasmids at 6.2 Mdal and 50 Mdal (Figure 1A). It was known that these strains belong to a group with similar plasmid patterns and had a high possibility of being borne by the plasmid [13].

It was assumed that the pathogenic gene, *psv*A associated with the establishment of canker disease, and the gene(s) involved in halo leaf spot formation, implicated that the plasmids of the NAE89 strain existed. Although the *psv*A gene was not cut by *Bam*HI, the plasmids of the NAE89 strain were extracted for the first time, cut by *Bam*HI, and used for agarose gel electrophoresis.

**Figure 2:**
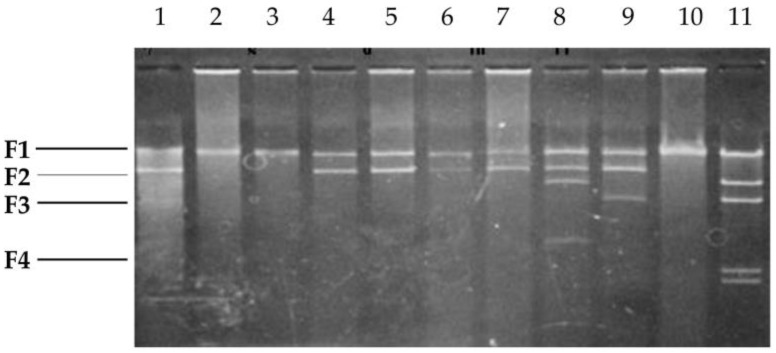
Size of DNA fragment of the pLAFR3 cosmid clone. Lane 1: NEA89 plasmid/*Bam*HI (F1–F4); Lane 2: pLAFC1/*Bam*HI (F1); Lane 3: pLAFC2/*Bam*HI (F1); Lane 4: pLAFC3/*Bam*HI (F2); Lane 5: pLAFC4/*Bam*HI (F2); Lane 6: pLAFC5/*Bam*HI (F2); Lane 7: pLAFC6/*Bam*HI (F2); Lane 8: pLAFC7/*Bam*HI (F2, F4); Lane 9: pLAFC8/*Bam*HI (F2, F3); Lane 10: pLAFC9/*Bam*HI (F1); Lane 11: standard of λ/*Hin*dIII.

Consequently, four DNA fragments—22.8 kb (F1), 12.3 kb (F2), 6.6 kb (F3), and 3.8 kb (F4)—were produced (Figure 1B). The fragment of λ/*Hin*dIII was used as a standard and its molecular weight used to calculate the size of each DNA fragment. After ligation via in vitro packaging, the plasmids of the pLAFR3 and NAE89 strains were digested with *Bam*HI and introduced into the *E. coli* strain DH5. All plasmids of the 200 obtained clones were examined (data not shown) based on their patterns and molecular weights. Their sizes became larger than pPLAFR3 in each plasmid, and the cloned pLAFR3 plasmids were identified (Figure 2). Furthermore, the plasmids derived from those clones were digested with *Bam*HI and agarose gel electrophoresis was performed. Investigations were undertaken on the clones of the *Bam*HI fragments of the observed NAE89 plasmid.

Results showed that pLAFC1, pLAFC2, and pLAFC9 were inserted into the F1 fragment at 22.8 kb. Whilst pLAFC3, pLAFC4, pLAFC5, and pLAFC6 were inserted into the F2 fragment at 12.3 kb, pLAFC8 was inserted into the F2 and F3 fragments, pLAFC7 was inserted into the F2 and F4 fragments, and a fragment of 9.4 kb was inserted into the *Bam*HI site of pLAFR3 (Figure 2). In addition, although the fragment of 9.4 kb was also newly inserted into pLAFC7, the F3 and F4 fragments were joined together.

Four NAE89 plasmid fragments digested with *Bam*HI were inserted. PE0 and NAE89-1, a bacterial strain non-pathogenic to the loquat, were introduced into NAE89 via triparental matings. Consequently, the inserted PE0 caused one deletion, and all fragment sizes become smaller. In the case of NAE89-1, the deletion occurred in two strains, while most were completely introduced. The result of a deletion varied between pLAFRC1 and pLAFRC2, in which F1 was the longest fragment inserted into PE0 (data not shown).

### 2.2. Pathogenic Experiment

**Figure 3:**
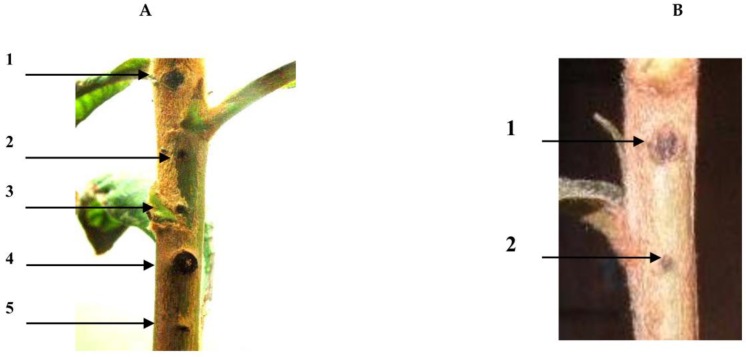
Pathogenic expression of each cloned plasmid inoculated to loquat stem. (**A**) Virulent expression: 1. PE0-TM (C2); 2. PE0-TM (C5); 3. PE0-TM (C8); 4. NAE6; 5. PE0-TM (C7); (**B**) Invirulent expression (controls): 1. PE0-TM (C1) (PE0 without insertion), (2) distilled water; TM: treatment.

The PE0 bacteria of the loquat did not carry the code for the *psv*A gene. As a result, the PE0 bacteria did not exhibit canker symptoms. Therefore, the NAE89 was a mutation stock that expressed a loss of halo leaf symptoms during its formation. The PE0 and NAE89-1 were introduced into pLAFR3, in which each *Bam*HI fragment of the NAE89 plasmid was inserted by triparental matings. pLAFC1 and pLAFC2 from the F1 fragment with the largest size were inserted, and showed pathogenicity in the loquat stem. Other inserted fragments (F2–F4) did not display pathogenicity in the loquat stem (Figure 3A,B).

### 2.3. Southern Hybridization

**Figure 4:**
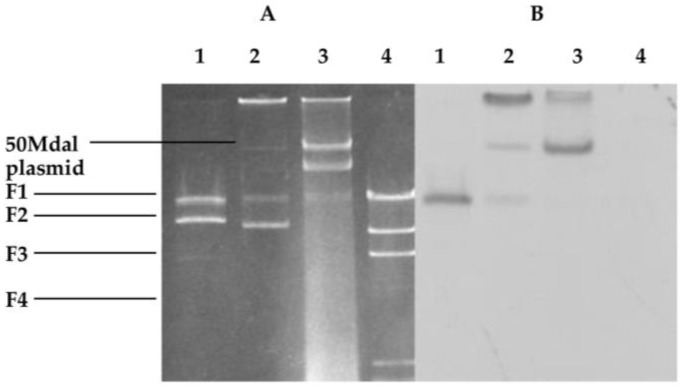
Southern hybridization of the NAE89 plasmid from the *P. syringae pathovar* using the virulence gene *psv*A as a probe. (**A**) Ethidium bromide gel. Lane 1: NAE89 was digested with *Bam*HI, lane 2: NAE89, lane 3: NAE6, lane 4: λ/*Hin*dIII as a standard; (**B**) Southern blot of gel in panel. The F1 fragment with the largest size was formed from hybridization.

The pathogenic examination revealed that the F1 fragment of the largest size showed pathogenicity. To investigate whether the pathogenic gene *psv*A existed, a Southern hybridization was performed using the pathogenic *psv*A (canker bacteria of the loquat) as a probe. Consequently, the F1 fragment hybridized with the largest size was formed, and the existence of the *psv*A gene(s) became clear (Figure 4B, lane 1). In addition, the 50 Mdal plasmid of the NAE89 strain and a hybrid were formed. The F1 fragment of the NAE89 obviously had a 50 Mdal origin (Figure 4A, lane 2). Of which, the 50 Mdal band of the plasmid was thin (unclear), and the hybrid was formed in the position of chromosomal DNA. This was part of the closed circular 50 Mdal fractured plasmid, and considered at a similar position as the linear chromosome using the agarose gel electrophoresis method. 

**Figure 5:**
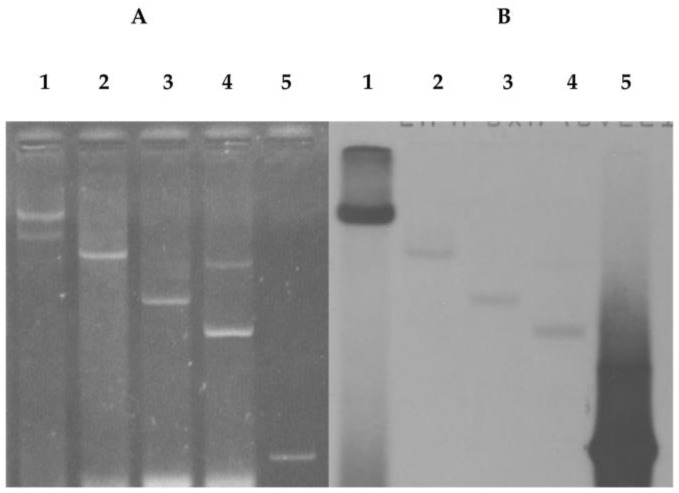
Southern hybridization showing that pathogenicity was recovered by a non-pathogenic strain in the triparental mating trial; (**A**) 1% agarose gel; (**B**) Southern blot of gel; Lane 1: NAE6; Lane 2: PE0-TM (C2) a; Lane 3: PE0-TM (C1) a; Lane 4: PE0-TM (C1) b; *psv*A used as a probe; TM (treatment).

However, the NAE6 strain was the 52 Mdal plasmid, from which the existence of the *psv*A gene was identified and hybrids were formed (Figure 4, lane 3). The pathogenicity was exhibited through a partial deletion when pLAFC1 and pLAFC2 were introduced into PE0 by triparental matings. It was proposed that there were deleted parts other than caused by the *psv*A gene(s), and the deletion plasmids introduced into PE0 were investigated by Southern hybridization. Each of these plasmids formed a hybrid with *psv*A, which indicated the existence of the *psv*A (Figure 5).

## 3. Discussion

This study showed that the strains of the group A harbored three plasmids at 25, 52, and 60 Mdal; the group B strains were at 6.2 and 50 Mdal; and the group C strains were at 32, 39, and 85 Mdal (Figure 1A). The present study showed that the *psv*A gene existed in the 50 Mdal plasmid of the NAE89 strain, which involved in stem canker formation. An attempt was made to detect a phenotypic marker that was encoded on the 52 Mdal plasmid in a similar manner [23]. By this study, it was found that the group B strain bacterium caused stem canker and halo leaf spots of loquat trees and harbored 6.2 and 50 Mdal gene fragments. 

Kamiunten [16] reported that the pLAFR3 cosmid clone, pVIR6, which contained a virulence gene in the 23 kb insert DNA region. This virulence gene originated from the 52 Mdal plasmid fragment of the group A strain *Ps. syringae* pv. *eriobotryae*. The deletion analyses of pVIR6 determined that about 7 kb of the inserted DNA restored pathogenicity to an avirulent PE0 strain. The plasmid in which the deletion occurred was denoted to be the pKPN35. A 6961 bp inserted to the DNA of pKPN35 was sequenced, and four possible open reading frames were found in tandem [16]. Of which, ORF1 and ORF4 exerted no significant homology to known genes. The ORF2 had an amino acid sequence similar to the transposase of the IS5 of *E. coli*. A recombinant plasmid pNSF1 containing only the ORF3 region restored pathogenicity to the avirulent PE0 strain. However, an ORF3 mutant of pNSF1, which was constructed by deleting a 580 bp *Bss*HII segment from ORF3, failed to restore virulence. Consequently, the ORF3 was identified as a virulent *psv*A gene [16].

In this experiment, it was found that the *Bam*HI fragment of the plasmid NAE89 strain, belonging to group B contained a known *psv*A virulence gene. The *Bam*HI cutting site and the halo leaf spot gene(s) being adjacent to the *psv*A gene of the cosmid vector pLAFR3 were also examined. Furthermore, the NAE89 strain plasmid produced four fragments (F1 fragment, 22.8 kb; F2 fragment, 12.3 kb; F3 fragment, 6.6 kb; F4 fragment, 3.8 kb). Following the result of investigating among 200 clones (data not shown), it was possible to obtain the *E. coli* DH5 into the four digested *Bam*HI fragments. Hence, each of *Bam*HI fragments (F1–F4) was introduced into NAE89-1 via triparental matings with PE0. When being inoculated (Figure 3), the F1 fragment of the largest size showed causal canker symptoms on the stems. 

This study was the first to provide evidence that the *psv*A gene associated with the 50 Mdal plasmid, and proposed that the 50 Mdal plasmid of the group B strain might play an important role in the development of canker symptoms on loquat stems and pathogenic activities. These findings provide useful information for the clarification of the halo leaf spot gene(s). Further trials with sequences should be carried out to re-estimate the positions of the observed fragments (F1–F4). In addition, the involvement of the *psvA* gene in the group B strain should be further investigated.

## 4. Materials and Methods

### 4.1. Bacterial Strains, Plasmids, Culture Conditions, and Chemicals

The bacterial strains and studied plasmids were listed in Table 1. The *Pseudomonas syringae* pv. *eriobotryae* strains were kindly supplied by Nagasaki Fruit-Tree Experiment Station, Nagasaki Prefecture, Japan. They were originally isolated from loquat stem cankers collected from various locations in Japan, and used as a probe in this study. The bacteria were ordinarily cultured in yeast extract–peptone (YP) medium (yeast extract 5 g, polypetone 10 g, NaCl 5 g, glucose 1 g, 1000 mL distilled water, pH 7.0) at 25 °C, following a method described in Hasebe et al. [24]. For growth on solid medium, 1.5% agar was added. *Escherichia coli* strains were grown in similar medium at 37 °C. *Hin*dIII-digested λ DNA was used as markers of standard molecular weight.

### 4.2. Plant Inoculation

Young yellow-green loquats were selected and washed in 70% ethanol to disinfect them of bacteria or fungi. Bacteria grown on YP agar (1.5%) medium for 24 h were inoculated into one-year-old loquat stems using a needle. The inoculated plants were placed in polyethylene bags for 12 h to keep the humidity high, then they were transferred to a growth chamber at 25 °C with a 12 h photoperiod. The symptom of halo spot formation developments on the stems was observed at 60 days after inoculation.

### 4.3. General DNA Manipulation and Clone Plasmid 

Plasmid extraction, restriction enzyme digestion, DNA ligation, plasmid clone, and agarose gel electrophoresis were carried out using standard procedures [22,25]. Plasmids were introduced into the *E. coli* by transformation and into *Ps. syringae* pv. *eriobotryae* by triparental matings with pRK2013 as a conjugative plasmid.

### 4.4. Southern Hybridization Experiments

The isolation of total genomic DNA was performed following a method previously described by Kamiunten [13]. The cosmid vector pLAFR3 was digested with *Bam*HI to convert to a single linear fragment and electrophoresed. The linearized pLAFR3 was then isolated from the agarose gel with an Easytrap Kit (Takara Biochemicals, Tokyo, Japan) and used as probe DNA to detect the integrated NAE6 into chromosomal DNA. To identify the virulence expression, the gene *psv*A (ORF3) was used as a hybridization probe. The labelling of probe DNA with horseradish peroxidase, hybridization and detection by exposure on autoradiography film were done using an ECL direct nucleic acid labelling and detection kit (Amersham, London, UK) (GE Healthcare Life Sciences, Little Chalfont, UK).

## Figures and Tables

**Table 1:** Bacterial strains and plasmids

Strains or Plasmids	Relevant Properties	Source or Reference
*Pseudomonas syringae* pv. *eriobotryae*		Nagasaki Fruit-Tree Experiment Station, Japan
NAE6	Wild-type virulence in group A	[13]
PE0	Plasmid lost NAE 6	[13]
NAE89	Wild-type virulence in group B	Nippongene
NAE89-1	The variant of lost halo gene formation	Nippongene
NAE57	Wild-type in the C group	Nippongene
*E. coli* DH5	*E. coli* bacteria and bacterium strain	Nippongene
pET-3a (*psvA*)	Vector inserted with *Bam*HI to *psv*A used as a probe in this study, isolated from a bacteria lesion of the strain group A	Stratagene
pRK2013	Helper plasmid	This study
pLAFR3	Cosmid vector	This study

## Supplementary Materials

Supplementary File 1
