# Supplementary material for: Association and Expression of Virulence from Plasmids of the Group B Strain in Pseudomonas syringae pv. eriobotryae
Source: Pathogens. 2018 Apr 14;7(2):41. doi: 10.3390/pathogens7020041 (PMC6027306; doi:10.3390/pathogens7020041)
Supplement: Supplementary File 1 [file pathogens-07-00041-s001.pdf]

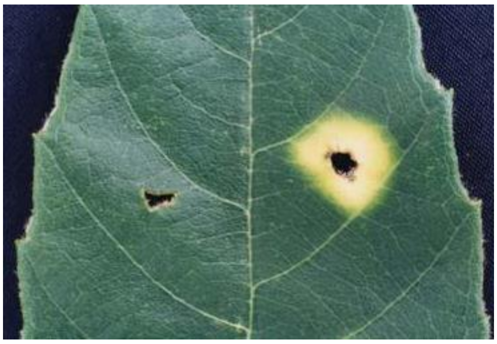

Supplement 1. Pathogenic expression observed on loquat leaves; Leaf: NAE6 (strain group A); Right: NAE87 (strain group B)

Molecular weight (Mdal)

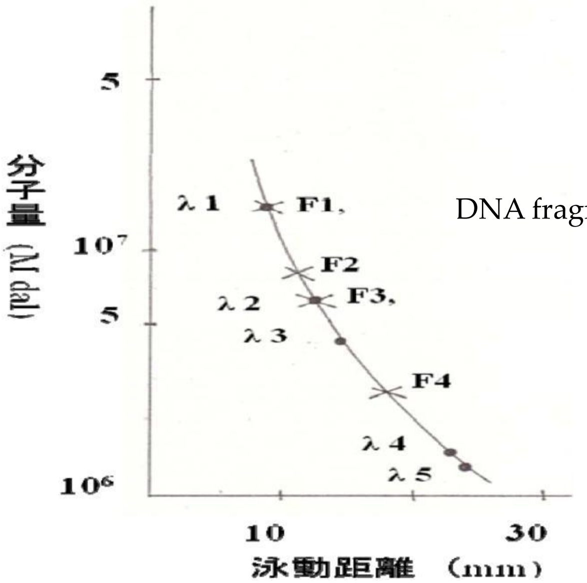

DNA fragments

Length of gel (mm)

Supplement 2. Correlation between DNA fragments and length of gel electrophoresis

pLAFR3 –

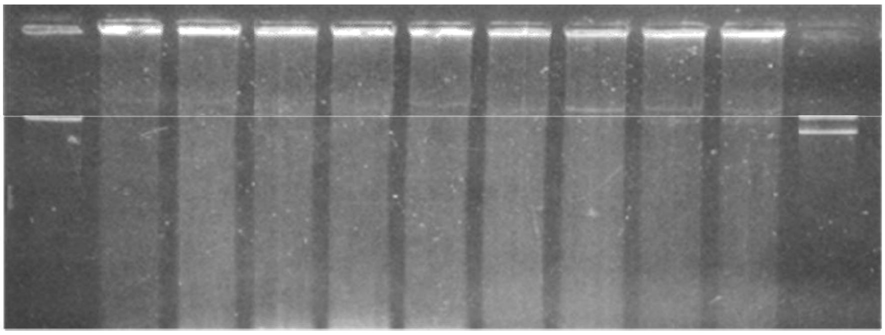

Supplement 3. Positions of the cloned NAE89/*Bam*HI plasmid from pLAFR3

1. pLAFR3
2. plAFRC1
3. pLAFRC2
4. pLAFRC3
5. pLAFRC4
6. pLAFC5
7. pLAFC6
8. pLAFC7
9. pLAFC8
10.pLAFC9
11. pLAFC10
